# Molecular Mechanism of Tocotrienol-Mediated Anticancer Properties: A Systematic Review of the Involvement of Endoplasmic Reticulum Stress and Unfolded Protein Response

**DOI:** 10.3390/nu15081854

**Published:** 2023-04-12

**Authors:** Kok-Lun Pang, Chun-Wai Mai, Kok-Yong Chin

**Affiliations:** 1 Department of Pharmacology, Faculty of Medicine, Universiti Kebangsaan Malaysia, Bandar Tun Razak, Cheras, Kuala Lumpur 56000, Malaysia; kok-lun.pang@newcastle.edu.my; 2 Newcastle University Medicine Malaysia, Iskandar Puteri 79200, Malaysia; 3 Department of Pharmaceutical Chemistry, Faculty of Pharmaceutical Sciences, UCSI University, Kuala Lumpur 56000, Malaysia; maicw@ucsiuniversity.edu.my

**Keywords:** ATF6, apoptosis, autophagy, ceramide, calcium, IRE1, PERK, vitamin E

## Abstract

Background: Tocotrienol, a type of vitamin E, is well known for its anti-cancer and other biological activities. This systematic review aims to summarize the involvement of endoplasmic reticulum stress (ERS) and subsequent unfolded protein response (UPR) as the underlying molecular mechanisms for the anticancer properties of tocotrienol. Method: A comprehensive literature search was performed in March 2023 using the PubMed, Scopus, Web of Science, and EMBASE databases. In vitro, in vivo, and human studies were considered. Result: A total of 840 articles were retrieved during the initial search, and 11 articles that fit the selection criteria were included for qualitative analysis. The current mechanistic findings are based solely on in vitro studies. Tocotrienol induces cancer cell growth arrest, autophagy, and cell death primarily through apoptosis but also through paraptosis-like cell death. Tocotrienol-rich fractions, including α-, γ- and δ-tocotrienols, induce ERS, as evidenced by upregulation of UPR markers and/or ERS-related apoptosis markers. Early endoplasmic reticulum calcium ion release, increased ceramide level, proteasomal inhibition, and upregulation of microRNA-190b were suggested to be essential in modulating tocotrienol-mediated ERS/UPR transduction. Nevertheless, the upstream molecular mechanism of tocotrienol-induced ERS is largely unknown. Conclusion: ERS and UPR are essential in modulating tocotrienol-mediated anti-cancer effects. Further investigation is needed to elucidate the upstream molecular mechanism of tocotrienol-mediated ERS.

## 1. Introduction

The endoplasmic reticulum (ER) is one of the essential intracellular organelles. It can be divided into rough and smooth ER, depending on the presence of ribosomes on the outer surface. Rough ER synthesizes proteins and subjects them to folding and post-translational modification [1]. Protein folding is essential for converting a polypeptide chain into a fully functional protein in a tertiary or three-dimensional structure [2]. Several factors, including extreme temperature, extreme pH, exogenous chemicals or denaturants, hypoxia, nutrient deprivation, viral infection, mechanical forces, aberrant calcium ion homeostasis, oxidative damage, or protein mutation, can cause interruption (unfolding) and/or errors in protein folding (misfolding) [3,4]. The accumulation of these unfolded or misfolded proteins in the ER lumen is known as ER stress (ERS), which triggers the unfolded protein response (UPR) to restore ER homeostasis and folding capacity [3,5].

**Figure 1:**
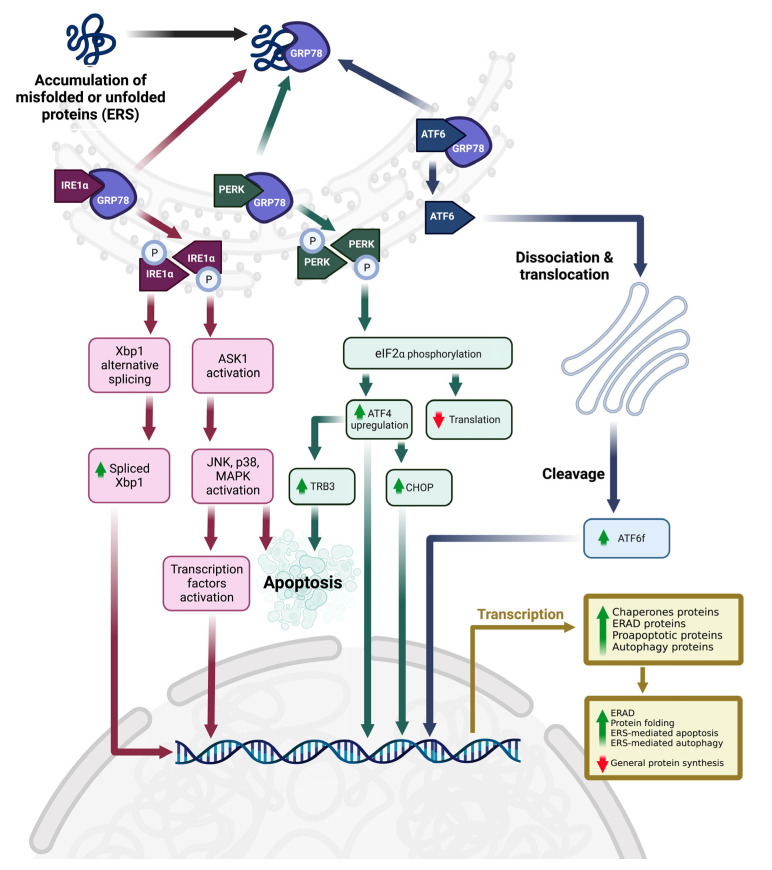
ERS, downstream UPR pathways, and cell fate. Abbreviations: ℗, phosphorylated; ASK1, apoptosis signal-regulating kinase 1; ATF4, activating transcription factor 4; ATF6, activating transcription factor 6; ATF6f, ATF6 fragment or cleaved form; CHOP, CAAT/enhancer-binding protein homologous protein; eIF2α, eukaryotic initiation factor 2 α subunit; ERAD, ER-associated degradation; ERS, endoplasmic reticulum stress; GRP78, 78 kDa glucose-regulated protein; IRE1α, inositol requiring element 1α; JNK, c-Jun N-terminal kinase; MAPK, mitogen-activated protein kinase; PERK, protein kinase-like endoplasmic reticulum kinase; TRB3, tribbles 3; Xbp1, X-box binding protein 1. The figure was created by authors using BioRender.com on 4 April 2023.

There are three major UPR downstream pathways, which involve protein kinase-like ER kinase (PERK), inositol-requiring element 1 (mainly α type, IRE1α), and activating transcription factor 6 α type (ATF6α) as ERS sensors or UPR markers (Figure 1) [3]. In the normal physiological state, all these 3 UPR markers are inactivated because they are bound by the ER-specific chaperone, 78 kDa glucose-regulated protein (Grp78, also known as binding immunoglobulin protein, BiP, or heat shock protein A5, HSPA5) [6]. During ERS, Grp78 is dissociated from these UPR markers to temporarily stabilize unfolded or misfolded proteins [6,7]. The unbound IRE1α and PERK are then homodimerized or oligomerized to trans-autophosphorylate themselves to be activated. Activated IRE1α can activate the mitogen-activated protein kinase (MAPK) pathways, such as c-Jun N-terminal protein kinase (JNK) and p38 MAPK, via the activation of apoptosis-signaling kinase 1 (ASK1, an upstream MAPK kinase kinase). However, activated IRE1α also induces X-box binding protein 1 (Xbp1) alternative splicing, and the spliced Xbp1 subsequently encodes a leucine zipper transcription factor with a transactivation domain, which could increase the expression of UPR downstream genes.

The activated PERK, on the other hand, phosphorylates downstream targets such as eukaryotic initiation factor 2 α subunit (eIF2α) to suppress global native protein synthesis [5]. At the same time, phosphorylated eIF2α also specifically increases the activating transcription factor 4 (ATF4) level, which subsequently increases the CAAT/enhancer-binding protein homologous protein level (CHOP) [8] and tribbles 3 (TRB3) [9]. CHOP is another transcription factor. Both ATF4 and CHOP specifically downregulate the antiapoptotic protein expressions [B-cell lymphoma 2 (Bcl-2) and B-cell lymphoma-extra large] and upregulate the expression of proapoptotic proteins [ER oxidoreductin 1 (ERO1), Bcl-2 interacting mediator of cell death, Bcl-2 associated X-protein, and Bcl-2 antagonist/killer protein] [8,10,11]. ERO1 is an oxidoreductase involved in ER’s reactive oxygen species (hydrogen peroxide) production, which causes calcium depletion in ER and further enhances apoptotic processing [8,12]. Furthermore, TRB3 is proapoptotic, whereby it inhibits the antiapoptotic activity of Akt and promotes the activation of caspases [8,13].

On the other hand, unbounded ATF6α is dissociated from ER and then translocated into the Golgi apparatus to be cleaved into a smaller cytosolic N-terminal ATF6 fragment (ATF6f) [3,5,14]. ATF6f is an active form of ATF6, whereby it translocates into the nucleus to induce UPR downstream gene expression via its transcriptional activation domain and DNA-binding domain [3,14]. As a result, UPR produces several essential proteins or enzymes to reduce general protein synthesis, increase protein chaperone levels to stabilize unfolded or misfolded proteins, induce ER-associated degradation (ERAD) of unfolded or misfolded proteins via proteasomal degradation or autophagy, or particularly induce ERS-mediated cell death when ERS is overwhelming [15].

**Figure 2:**
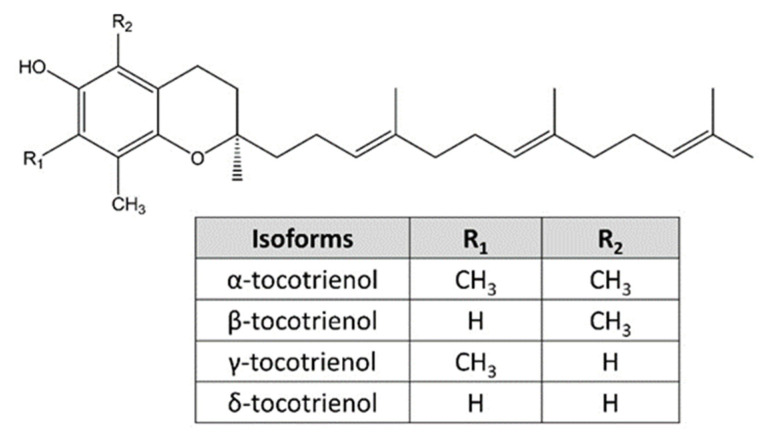
Four isoforms of tocotrienol and their chemical structures.

Bioactive compounds from natural resources possess great potential in novel drug design and development. Tocotrienol and tocopherol are two natural vitamin E families. Structurally, tocopherol and tocotrienol consist of a chromanol ring and a carbon tail. The distinction is that tocopherol has a saturated hydrocarbon phytyl tail and tocotrienol has an unsaturated farnesyl tail with three carbon-carbon double bonds [16,17,18]. Tocopherol and tocotrienol can be further classified into 4 isoforms, namely α-, β-, γ-, and δ-tocotrienol, due to the variation in the position of side chains on the chromanol ring (Figure 2) [17,19]. Tocotrienols are found naturally in vegetable oils, including palm oil, rice bran oil, barley oil, and annatto seed oil, and in trace amounts in sunflower seed, grapefruit seed, nuts, vegetables, and fruits [20,21,22].

Tocotrienol demonstrates broad biological activities, including antioxidant, anti-inflammatory, cardioprotective, neuroprotective, anti-metabolic, anti-osteoporotic, anti-rheumatic, and anti-diabetic properties [23,24,25,26]. Tocotrienol is known to inhibit 3-hydroxy-3-methyglutaryl-CoA (HMG CoA) reductase activity and expression to exert cholesterol-lowering effects [27,28,29]. Additionally, tocotrienol is widely reported to have anti-cancer properties, whereby it induces growth arrest and cell death, primarily apoptosis, in several cancerous cells originating from the mammary gland, digestive tract, liver, prostate, lung, and bone cancer [24,30,31,32,33,34,35]. Mechanistically, the anticancer properties of tocotrienol are subjected to the types of isoforms and cancer cell models. Tocotrienol-mediated anticancer effects involved the inhibition of several signaling and transduction pathways, such as signal transducer and activator of transcription 3 (STAT3), wingless and integrated/glycogen synthase kinase 3β, phosphoinositide 3-kinase/Akt, HMG CoA reductase, Ras/Raf/MAPK kinase/MAPK, and nuclear factor kappa B pathways [36]. Additionally, tocotrienol was reported to induce ERS activities, which are essential in exerting its biological activities such as neuroprotection [37] and anti-cancer [38,39]. To our knowledge, a review that specifically and systematically summarizes the ERS-inducing properties of tocotrienol and its molecular mechanisms is not available. The effects of tocotrienol were reviewed along with those of other natural products with ERS-inducing properties 4–5 years ago [38,39]. We are speculating that more studies on this topic have been published since then. Therefore, in this systematic review, we aim to systematically summarize the anti-cancer effects of natural isoforms of tocotrienols, particularly the involvement of ERS and UPR and their upstream molecular mechanisms.

## 2. Materials and Methods

This systematic review was conducted according to preferred reporting items for systematic reviews and meta-analyses (PRISMA) guidelines (Supplementary Table S1) [40]. The protocol of this systematic review was registered in the Open Science Framework (access link: https://doi.org/10.17605/OSF.IO/B4KAQ, registered on 15 March 2023). In addition, four databases, including PubMed, Scopus, Web of Science, and EMBASE, were used for literature searching in March 2023. The following search terms were used: (1) (“vitamin E” OR tocotrienol) and (2) (“endoplasmic reticulum stress” OR ERS OR “unfolded protein response” OR UPR). Additional records were identified manually from the reference list of included research articles or relevant review articles. The detailed search strategy is listed in Supplementary Table S2.

**Figure 3:**
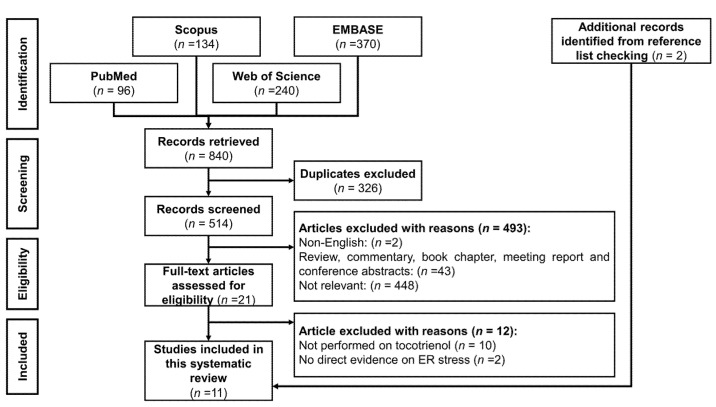
PRISMA flow chart of the systematic review.

The inclusion criteria for this systematic review were studies that reported tocotrienol-mediated ERS or UPR and the associated molecular mechanism. There is no restriction on the year of publication or study model (including in vitro, in vivo, and human). The exclusion criteria were: (1) articles not written in English; (2) articles without primary data; (3) studies using semi-synthesized or modified tocotrienol; and (4) combinational therapy, whereby the effects of tocotrienol treatment alone could not be delineated. The article identification and screening process was summarized in Figure 3.

Two independent reviewers (K.-L.P. and C.-W.M.) extracted the data and performed the screening of the titles and abstracts, followed by the screening of the full-text articles. Inclusion and exclusion criteria were applied, and any discrepancy between reviewers was resolved by discussing it with the third author (K.-Y.C.). The extracted information was summarized in an evidence table (Table 1), including the name of the first author, year of publication, treatment, study model, major findings, and quality assessment.

Two independent reviewers (K.-L.P. and C.-W.M.) evaluated the quality of included articles by using the Office of Health Assessment and Translation (OHAT) risk of bias tool for non-human studies [41]. Nine domains were evaluated, including “Randomization,” “Allocation concealment,” “Experimental condition,” “Blinding during experiment,” “Incomplete or missing data,” “Exposure characterization,” “Outcome assessment,” “Completeness of reported outcomes,” and “Other consideration” [42]. The included articles were assessed and graded into Tier 1 (high quality), Tier 2 (moderate quality), and Tier 3 (low quality), as previously reported [42]. Disagreement between reviewers was resolved by discussion among all authors. The quality of articles was included in Table 1, and the breakdown of the OHAT assessment was shown in Supplementary Table S3.

**Table 1:** Summary of information and findings of the included studies.

Authors	Treatment	Models	Major Findings	OHAT Tier
Wali et al. [43]	γ-tocotrienol (0–40 μM, 24 h treatment)	Mammary tumor +SA cells	γ-tocotrienol induced concentration- and time-dependent cell death with the upregulation of ERS response signaling proteins (phosphor-PERK, phosphor-eIF2α, ATF4, ATF6α), cleaved caspase-12 and ERS-related cell death proteins (CHOP & TRB3, but not Grp78). Tocotrienol-mediated ERS was independent of Grp78 and the mevalonate pathway.	1
Park et al. [44]	α-, δ- & γ-tocotrienols (0–40 μM, 24 h treatment)	Murine mammary tumour 66cl-4-GFP cells, human mammary tumour MCF-7, MDA-MB-231 and MDA-MB-468 cells	δ- & γ-tocotrienols exerted potent anti-cancer activities as compared to α-tocotrienol. γ-tocotrienol induced mammary tumor cell apoptosis in JNK- & p38-mediated CHOP and DR5-dependent manner. ERS was involved in the upstream mechanism of tocotrienol-induced apoptosis as evidenced by the upregulation of ATF4, CHOP, and Grp78 levels; and Xbp-1 mRNA splicing. ERS inhibitor (salubrinal) protected the cells from γ-tocotrienol-induced MAPK activation and apoptosis.	1
Gopalan et al. [45]	γ-tocotrienol (0–10 μM, 24–72 h treatment)	Human mammary tumor MCF-7 and MDA-MB-435 cells	γ-tocotrienol was more potent than γ-tocopherol. γ-tocotrienol induced mammary tumor cell apoptosis with caspases activation, PARP cleavage, JNK activation, and upregulation of DR5 and CHOP levels. γ-tocotrienol increased the intracellular ceramide and dihydroceramide levels. De novo ceramide synthesis inhibitor protected the cells from tocotrienol-mediated apoptosis, JNK activation, DR5 and CHOP upregulation, and caspases activation.	1
Patacsil et al. [46]	α- & γ-tocotrienols (0–80 μM, 24–72 h treatment)	Human mammary tumor MCF-7 and MDA-MB-231 cells, and non-cancerous human mammary MCF-10A cells	γ-tocotrienol was more potent than α-tocotrienol. γ-tocotrienol induced mammary tumor cell G1 arrest and apoptosis. Transcriptomic analysis revealed the involvement of ERS response and UPR pathways. γ-tocotrienol upregulated the Grp78, ATF3 and CHOP levels with ERS markers (ATF4, phosphor-PERK, phosphor-IRE1α & eIF2α but not ATF6).	1
Xiong et al. [47]	γ-tocotrienol (0–20 μM, 24 h treatment)	Human mammary tumor MDA-MB-231 and SUM159 cells	γ-tocotrienol induced mammary tumor cell apoptosis with the upregulation of Grp78, CHOP & DR5 levels.	1
Tuerdi et al. [48]	γ-tocotrienol (20 μM, 24–48 h treatment)	Human malignant mesothelioma H2052, H28, H242 and MSTO-211H cells	γ-tocotrienol induced malignant mesothelioma cell death with the increase in CHOP, Grp78, and caspase-4 mRNA levels.	1
Tiwari et al. [49]	γ-tocotrienol (40 μM, 6–24 h treatment)	Human mammary tumour MCF-7 and MDA-MB-231 cells, and non-cancerous human mammary MCF-10A cells	γ-tocotrienol induced mammary tumor cell apoptosis and autophagy with JNK & p38 (but not ERK) activation and early upregulation of Grp78, TRB3, CHOP and ERS markers (IRE1α, phosphor-PERK, phosphor-eIF2α ATF4).	1
Comitato et al. [50]	TRF, α-, δ- & γ-tocotrienols (5–20 μg/mL, 24–48 h treatment) * 12.6–50.4 μM (δ-tocotrienol) and 12.2–48.7 μM (γ-tocotrienol)	Human cervical tumour HeLa cells and human mammary tumour MCF-7 cells without oestrogen receptor	α-, δ- & γ-tocotrienols (but not TRF) induced the release of endoplasmic reticulum calcium ions into the cytosol. δ- & γ-tocotrienols upregulated the Xbp-1 and CHOP mRNA levels, upregulated Grp78 protein level, and ERβ-independent Xbp-1 alternative splicing and caspase-12 activation. Tocotrienols (especially δ-tocotrienol) induced IRE1α phosphorylation but not ATF6 and PERK phosphorylation.	1
Marelli et al. [51]	δ-tocotrienol (5–20 μg/mL, 24–48 h treatment) * 12.6–50.4 μM	Human melanoma BLM and A375 cells, and human primary melanocytes Melanoma-xenograft nude mice model was used but no contribution to the mechanistic findings	δ-tocotrienol induced cytotoxicity and apoptosis on melanoma cells but not on non-cancerous melanocytes. δ-tocotrienol activated the caspase 4 and upregulated the ERS markers (Grp78, PERK, phosphor- eIF2α & IRE1α) and ERS-related apoptosis markers (ATF4, CHOP & ERO1α). δ-tocotrienol induced nuclear translocation of CHOP and ATF4 and upregulated the CHOP and IRE1α mRNA. Salubrinal protected the melanoma cells from δ-tocotrienol-induced ERS-mediated apoptosis.	1
Fontana et al. [52]	δ-tocotrienol (0–20 μg/mL, 24–72 h treatment) * 0–50.4 μM	Human prostate tumour DU145 and PC3 cells, and non-cancerous human prostate epithelial RWPE-1 cells	δ-tocotrienol induced cytotoxicity, apoptosis and autophagy on prostate cancer cells but not on non-cancerous melanocytes. δ-tocotrienol upregulated ERS markers (Grp78, phosphor-eIF2α & IRE1α) and ERS-related apoptosis markers (ATF4 & CHOP). Salubrinal and 4-phenylbutyrate protected the prostate tumour cells from δ-tocotrienol-induced ERS-mediated apoptosis and autophagy.	1
Ambra et al. [53]	δ- & γ-tocotrienols (5–20 μg/mL, 24 h treatment) * 12.6–50.4 μM (δ-tocotrienol) and 12.2–48.7 μM (γ-tocotrienol)	Human cervical tumour HeLa cells	γ-tocotrienol significantly upregulated 3 miRNAs including miR-190b, miR-215 and miR-148a. δ- & γ-tocotrienols induced Xbp1 alternative splicing via miR-190b. Anti-miR-190b suppressed while miR-190b overexpression promoted tocotrienol-induced apoptosis.	1

* Tocotrienol concentration was converted based on the molecular weight of 410.6 g/mol (γ-tocotrienol) and 396.6 g/mol (δ-tocotrienol). Abbreviations: ATF3, activating transcription factor 3; ATF4, activating transcription factor 4; ATF6, activating transcription factor 6; CHOP, CAAT/enhancer-binding protein homologous protein; eIF2α, eukaryotic initiation factor 2 α subunit; ERK, extracellular signal-regulated kinase; ERO1α, ER oxidoreductin 1α; ERS, endoplasmic reticulum stress; Grp78, 78kDa glucose-regulated kinase; IRE1 α, inositol requiring element 1 α subunit; JNK, c-Jun N-terminal kinase; MAPK, mitogen-activated protein kinase; OHAT, Office of Health Assessment and Translation; PERK, protein kinase-like endoplasmic reticulum kinase; TRB3, tribbles 3; TRF, tocotrienol-rich fraction; Xbp1, X-box binding protein 1.

## 3. Results

### 3.1. Selection of Articles

A total of 840 records (96 articles from PubMed, 134 articles from Scopus, 240 articles from the Web of Science, and 370 articles from the EMBASE database) were identified. After removing the duplicates, 514 articles were screened for titles and abstracts. A total of 493 articles were excluded due to language (*n* = 2), lack of primary data (*n* = 43), and being irrelevant to the topic (*n* = 448). After assessing the eligibility of the full-text articles, 12 articles were excluded because 10 were not studying tocotrienol and 2 were using combination treatment, and the effects of tocotrienol treatment could not be determined. Two additional articles were included, and a final 11 articles were included for qualitative analysis in this systematic review.

### 3.2. Study Characteristics

The included articles were published between 2009–2020. All the included articles were in vitro studies. We found 2 animal studies [44,51], but they were not included in the qualitative analysis since they did not report the mechanism of action of tocotrienol. Mammary tumor cell lines were the most commonly used in vitro cancer cell models [43,44,45,46,47,49], followed by cervical cancer HeLa cells [50,53] and other cancer cell lines such as mesothelioma [48], melanoma [51], and prostate cancer [52] cell lines.

Tocotrienol rich-fraction (TRF) [50] or tocotrienol pure isoforms including α- [44,46,50], γ- [43,44,45,46,47,48,49,50,53], and δ-tocotrienol [44,50,51,52,53] were tested in the included studies. β-tocotrienol has not been studied, and we speculate that is because it is a less commonly available tocotrienol isoform [54]. The quality of all included articles was high (Tier 1) according to the OHAT classification [42]. However, all the included articles did not describe the experimental blinding. Tocotrienol stock solutions were prepared by dissolving it in sterile 10% bovine serum albumin solution [43,49], ethanol [44], dimethyl sulfoxide (DMSO) [46,50,51,53], a DMSO/ethanol mixture [45,47], or olive oil [51]. Two of the included studies did not disclose the tocotrienol stock preparation and/or storage [48,52]. Most of the included studies did not disclose the final concentrations of the vehicle (solvent) used [43,49,50,51,52,53]. Concentrated tocotrienols (≥95% purity) were used in most studies, but 4 studies did not disclose the purity of tocotrienol [44,45,47,48]. The tested tocotrienol concentrations were between 10–80 μM, and the treatment timeframe was typically 24 h [43,44,45,47,48,49,50,51,52,53]. Other treatment durations, such as 1 h [51], 6 h [49], 48 h [45,48,50,51], and 72 h [45,46,52], have been used.

Several ERS inducers were used in previous studies as comparative controls, including tunicamycin [43,46,50], thapsigargin [43], and brefeldin [50]. One study claimed the use of tunicamycin and brefeldin as controls, but the results for both controls were not shown [53]. Several studies used ERS inhibitors such as salubrinal [44,51] or 4-PHA [50,52] to confirm the role of ERS in cell death and/or autophagy. Carbachol was selected as a positive control to induce ER calcium ion release into the cytoplasm [50]. Myriocin and C8-cyclopropenylceramide, both known de novo ceramide synthesis inhibitors, were used to inhibit ceramide synthesis [45]. A study that did not employ any ERS inducer or inhibitor in their experiment [49]. Additionally, the combinational effects of tocotrienol with docosahexaenoic acid [47] and with simvastatin and atorvastatin [48] have been studied, but the current review would only focus on the effects of tocotrienol as a single agent.

### 3.3. Anti-Cancer Properties of Tocotrienols

From the included articles, tocotrienols demonstrated superior selectivity on cancerous cells and were not toxic to several non-cancerous cells within the anticancer concentration range, including human mammary tissue MCF-10A [46,49], human primary melanocytes [51], and prostate epithelial RWPE-1 cells [52]. Tocotrienols, including TRF, α-, δ- and/or γ-tocotrienols, significantly induced cancer cell growth arrest [46,48], autophagy [49,52], and cell death, mainly apoptosis [43,44,45,46,47,48,49,50,51,52], followed by paraptosis with extensive cytoplasmic vacuolation [52]. The molecular mechanisms of tocotrienol-mediated apoptosis involved the upregulation of pro-apoptotic mitochondrial proteins [48,49,51], initiator and executioner caspase activation [44,45,46,48,49,50,51,52], and PARP cleavage [43,44,45,46,49,51,52]. Further, γ-tocotrienol induced the extrinsic pathway of apoptosis on mammary tumor cells by upregulating death receptor 4 (DR4) and/or 5 (DR5) [44,45,47]. Wali et al., however, reported that DR5 expression was not changed on γ-tocotrienol-treated mouse malignant mammary tumor +SA cells [43].

### 3.4. ERS, UPR, and Upstream Molecular Mechanism

Tocotrienol-mediated ERS was demonstrated by activation of caspase-4 [51] or caspase-12 [43,50], upregulation of caspase-4 mRNA [48], or upregulation of ERS markers or sensors, including phosphor-PERK, phosphor-IRE1α, and ATF6α [43,46,49]. Tocotrienol-mediated PERK activation subsequently phosphorylated eIF2α and then upregulated ATF4 protein levels [43,44,46,49,51,52]. However, tocotrienol-mediated IRE1α activation triggered estrogen receptor-independent [50] Xbp1 alternative splicing [44,50]. Tocotrienol also upregulated the ERS-related apoptosis protein levels or mRNA expression of Grp78, CHOP, TRB3, and/or ERO1 [43,44,45,46,47,48,49,50,51,52]. Marelli et al. also reported that tocotrienol upregulated *CHOP* and *IRE1* mRNA and demonstrated the nuclear translocation of CHOP and ATF4 proteins [51].

MAPK signaling, including JNK and p38 MAPK, was activated after tocotrienol-mediated ERS. Tocotrienol activated p38 MAPK [44,49,52] and JNK, and subsequent c-Jun transcription factor, a JNK downstream substrate [44,49,52]. However, another MAPK member, extracellular signal-regulated kinase (ERK), was not activated. As evidenced by the decrease in its phosphorylated form upon tocotrienol treatment in a time-dependent manner [48,49]. Pre-treatment with salubrinal or 4-PBA (ERS inhibitors) significantly suppressed the tocotrienol-mediated MAPK activation and subsequent ERS-mediated apoptosis [44,50,51,52]. Interestingly, tocotrienol-mediated cell death was found to be independent of the mevalonate pathway, whereby mevalonate and its intermediate products, such as farnesyl pyrophosphate and geranylgeranyl pyrophosphate, did not protect the cells from γ-tocotrienol-mediated cell death [43,48]. This finding suggests an additional mechanism of action for tocotrienol apart from its HMG CoA reductase inhibition.

The upstream molecular mechanism of tocotrienol-induced ERS is largely unknown. Comitato et al. demonstrated α-, δ- & γ-tocotrienols induced an early ER calcium ion release into the cytoplasm as early as 15 min, which serves as the early signal in triggering ERS [50]. The released calcium ions could activate calpain and caspase-12, which subsequently activate ERS-mediated apoptosis [50]. On the other hand, Ambra et al. recently reported the role of miRNAs in regulating Xbp1 alternative splicing via δ- & γ-tocotrienol-mediated ERS [53]. δ- & γ-tocotrienol significantly upregulated miR-190b, miR-215, and miR-148a on HeLa cells. In silico analysis further suggested the highly potential interaction and complex formation between miR-190b and Xbp1. Subsequently, miR-190b inhibition and its overexpression confirmed the essential role of miR-190b in mediating δ- & γ-tocotrienol-induced Xbp1 alternative splicing. Lastly, accumulated evidence also suggests the role of ceramide in mediating tocotrienol-induced apoptosis. Gopalan et al. reported that γ-tocotrienol upregulated the intracellular ceramide and dihydroceramide (C16, C24, and total levels) [45,55]. Suppression of ceramide synthesis by myriocin and C8-cyclopropenylceramide suppressed the tocotrienol-mediated extrinsic pathway of apoptosis, upregulation of CHOP, and JNK activation [45]. Similarly, Xiong et al. [47] and Park et al. [44] also postulated the essential role of ceramide and its signaling pathway in tocotrienol-mediated ERS, but no relevant data was available to support their postulation.

## 4. Discussion

### 4.1. Tocotrienol-Induced ERS and ERS-Related Cell Death

The current finding summarizes the anticancer properties of tocotrienol by triggering ERS, UPR, and ERS-related apoptosis or autophagy on several cancerous cells. Interestingly, tocotrienol-mediated ERS and ERS-mediated apoptosis were not dependent on HMG CoA reductase inhibition [43,48]. This contradicts previous studies, where the combination of tocotrienol with other HMG CoA reductase inhibitors (such as statins) produced a synergistic effect in growth arrest and apoptosis [56,57,58]. Tocotrienol-mediated ERS and ERS-mediated apoptosis are independent of estrogen receptor signaling [50]. This is coherent with the previous studies, which reported that the anti-cancer properties of tocotrienols, including TRF, α-, γ- and/or δ-tocotrienol, were not dependent on estrogen receptor status [59,60]. Further study is required to confirm the role of HMG CoA reductase and estrogen receptor signaling in tocotrienol-mediated ERS and cell death.

Additionally, δ-tocotrienol also induced prostate tumor cell paraptosis with extensive cytoplasmic vacuolation [52]. A similar finding was reported, whereby annatto tocotrienol, γ- and δ-tocotrienols induced paraptosis or paraptosis-like cell death with extensive cytoplasmic vacuolation in osteosarcoma SW1353 cells [31], human melanoma A375 cells [61], and human colon cancer SW620 cells [62,63]. Furthermore, pre-treatment with salubrinal (anERS inhibitor) significantly suppressed δ-tocotrienol-mediated cytoplasmic vacuolation [52], which further suggests the importance of ERS in executing paraptosis. How tocotrienol-induced ERS leads to paraptosis processing is unknown, but it could be related to MAPK activation [64].

### 4.2. Contradicting Findings in Tocotrienol-Mediated ERS

Tocotrienol, especially γ-tocotrienol, specifically induced the extrinsic pathway of apoptosis, as evidenced by the upregulation of death receptors [44,45,47]. This observation contradicts the findings of Wali et al., whereby DR5 expression was not changed in γ-tocotrienol-treated mouse malignant mammary tumor +SA cells [43]. The tocotrienol-mediated extrinsic pathway of apoptosis might rely on the optimal treatment of tocotrienol. DR4 and DR5 were prominently upregulated upon 24 h of tocotrienol treatment with a concentration of 30 µM and above [44]. A 24 h-exposure to tocotrienol at 20 µM and below was suboptimal and could not upregulate DR5 [43,44]. A lower concentration of tocotrienol (10 µM) but a longer treatment time (72 h) could upregulate the DR5 in both MCF-7 and MDA-MB-435 cells [45], suggesting tocotrienol induced DR5 activity in a concentration- and time-dependent manner. Furthermore, Grp78 protein expression on tocotrienol-treated cancer cells was heterogenous (either upregulated or unchanged). It is speculated that an optimal treatment time and/or concentration would be required to demonstrate the upregulation of the Grp78 level, which is very similar to the reported death receptor expression. For instance, the Grp78 level was unchanged upon γ-tocotrienol treatment along with +SA cells (20 µM up to 24 h; 30 µM up to 8 h) [43]. A higher concentration of γ-tocotrienol treatment (24.4 µM for 24 h [50], 40 and 80 µM for 24 h [46], 40 µM for 24 h [47,49]) or δ-tocotrienol (15 & 20 µM for 16 h [44], 25.2 µM for 24 h [50], 37.8 µM for 18 h [52] or 50.4 µM for 16 h [51]) significantly upregulated Grp78 level on cervical cancer HeLa cells, melanoma BLM and A375 cells, or mammary tumor MCF-7 and MDA-MB-231 cells.

Furthermore, tocotrienol was reported to activate some but not all ERS sensors or UPR markers. Wali et al. demonstrated that γ-tocotrienol (20 µM, as early as 4 h) significantly increased the phosphorylated PERK and ATF6 degradation in +SA cells [43]. A 24h γ-tocotrienol (40 and/or 80 µM) also strongly induced the phosphorylation of PERK on MCF-7 and MDA-MB-231 cells [46,49], but without activating the ATF6 pathway [46]. In addition, a lower concentration (around 25 μM) of δ-tocotrienol (but not α- or γ-tocotrienol) for 24 h significantly induced IRE1α phosphorylation on HeLa cells [50]. However, none of these tocotrienol isoforms altered ATF6 and total PERK expression on HeLa cells [50]. Under its normal state, Grp78 is bound by inactive PERK, IRE1, and ATF6. During ERS, PERK, IRE1, and ATF6 are activated upon dissociation of Grp78, whereby Grp78 works as a chaperone to bind with unfolded or misfolded proteins in ER [7]. Nevertheless, the binding affinities of Grp78 with these ER sensors may vary and be highly subject to different physiological conditions [65]. This may partially explain the specific activation of particular ER sensors upon tocotrienol treatment. Other factors, such as tocotrienol treatment conditions, isoforms, cell models, and the detection limit of the assay (for instance, Western blotting), may also contribute to the heterogeneous findings.

### 4.3. Novel Approaches in Studying Tocotrienol-Mediated ERS

Several transcriptomic analyses revealed the involvement of ERS and/or UPR response pathways upon tocotrienol treatment [31,46,66]. γ-tocotrienol (40 μM for 24 h) significantly upregulated the mRNAs of ERS-modulated genes including *ATF3*, *CHOP*, *PERK,* and *GRP78* [46]. Annatto tocotrienol (28.5 μg/mL), δ- (49.2 μM), and γ-tocotrienols (93.8 μM) treatment for 24h also significantly triggered several pathways, such as “Response to ERS,” “UPR,” and “Protein processing in ER” [31]. Similarly, another transcriptomic analysis on astrocytoma and glioblastoma cells with the combinational treatment of γ-tocotrienol (97.4 μM) and hydroxychavicol also revealed the involvement of the “UPR pathway,” “UPR signaling protein activity,” and “ERS-related intrinsic pathway of apoptosis” [66]. Despite the potential of the high-throughput transcriptomic approach, it is noteworthy that ATF6 degradation and phosphorylation of PERK or IRE1 are the established approaches for defining ERS [5,67]. Additionally, the level of total cleaved ATF6 (50 kDa) or nuclear translocation of these cleaved ATF6 can be employed to indicate the activation of the ATF6 pathway [67,68]. ER dilation (as detected by electron microscopy) and ER redox status (by ER-targeted redox-sensitive green fluorescent protein) upon tocotrienol treatment are also useful in identifying ERS formation [5,67]. Some of the included studies [49,51,52] only examined the total IRE1α or PERK level without their phosphorylated forms, which may lead to interpretation bias.

MicroRNAs are a type of short noncoding RNAs (~22 nucleotides) important in regulating cellular response and function, including UPR [69,70]. Several miRNAs such as miR-30d [71,72], miR-199a-3p or miR-199a-5p [73], miR-214 [73], miR-379-5p [71,72], and miR-7112-3p [74] were reported to target Grp78, thus affecting protein folding and leading to ERS. The understanding of the involvement and role of miRNA in tocotrienol-mediated ERS is very limited. Recently, miR-190b was found to be crucial in executing Xbp1 alternative splicing in δ- & γ-tocotrienol-mediated ERS [53]. Upregulation of miRNA can be due to an increase in DNA copy number, activation of transcription factors, or DNA/RNA methylation [75]. The mechanism of miR-190b upregulation upon tocotrienol treatment, however, has not been determined.

### 4.4. Current Understanding of the Upstream Molecular Mechanisms of Tocotrienol-Mediated ERS

The upstream molecular mechanism of tocotrienol-induced ERS, including the activation of PERK and IRE1, is largely unknown. All the included studies only demonstrated the activation of UPR markers; the accumulation of misfolded or unfolded protein upon tocotrienol treatment has not been determined. In addition, how tocotrienol treatment triggers the activation of UPR markers is not fully understood. Tocotrienol induces very early ER calcium ion release, which suggests that it serves as an early signal in triggering ERS [50]. Glucose or energy deprivation serves as a physiological ER inducer by disrupting cellular energy production and ER calcium ion homeostasis [76]. Tocotrienol was demonstrated to suppress aerobic glycolysis and ATP production in human mammary tumor MDA-MB-231 cells [77]. Nevertheless, energy deprivation does not explain the very rapid release of ER calcium upon tocotrienol treatment because it generally takes a much longer time (around 24h) to induce ERS [67]. Several compounds, such as thapsigargin, a known non-competitive sarcoplasmic/ER calcium ion ATPase pump (SERCA) inhibitor [78], inhibit the transfer of cytosolic calcium ions into the ER lumen, which subsequently leads to ER calcium depletion, ER chaperone loss of activity, and eventually the accumulation of unfolded protein [67,78]. Interestingly, similar to thapsigargin, high levels of sphingolipids such as ceramide also induce ERS by inhibiting SERCA [79]. Tocotrienol-mediated anti-cancer effects (especially γ-tocotrienol) were demonstrated through an increase in intracellular ceramide and/or dihydroceramide levels [45,55,80]. The upregulation of dihydroceramide upon δ-tocotrienol treatment, for instance, was reported to upregulate the phosphorylation of JNK, eIF2α, and the inhibitor of kappa B α kinase, which subsequently contributes to ERS-dependent anti-inflammation [81]. Exogenous ceramide also induces ERS-mediated apoptosis via a p38 MAPK-and JNK-dependent manner [82], which is very similar to tocotrienol-mediated ERS. Mechanistically, γ-tocotrienol is reported to inhibit dihydroceramide desaturase in human pancreatic cancer PANC-1, colorectal cancer HCT-116, and MCF7 cells, which indirectly leads to the accumulation of dihydroceramide [83]. Direct stimulation of ceramide synthesis is also postulated, but more direct evidence is needed [45,55,80,83]. Further, γ-tocopherol with a saturated phytyl tail also significantly increased the ceramide and dihydroceramide levels [45,55], which indicates the potential role of the chromanol ring but not the hydrocarbon tail in regulating ceramide or dihydroceramide levels.

**Figure 4:**
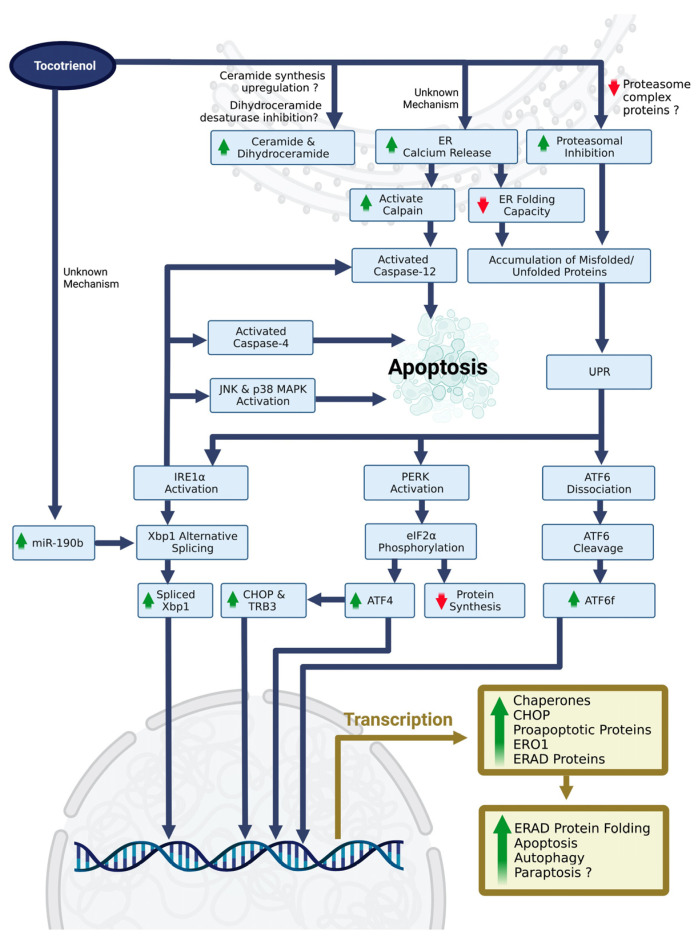
Reported and postulated molecular mechanisms of tocotrienol-mediated ERS and ERS-related cell fate. Abbreviations: ATF4, activating transcription factor 4; ATF6, activating transcription factor 6; ATF6f, ATF6 fragment or cleaved form; CHOP, CAAT/enhancer-binding protein homologous protein; eIF2α, eukaryotic initiation factor 2 α subunit; ER, endoplasmic reticulum; ERAD, ER-associated degradation; ERO1, ER oxidoreductin 1; IRE1α, inositol requiring element 1α; JNK, c-Jun N-terminal kinase; MAPK, mitogen-activated protein kinase; PERK, protein kinase-like endoplasmic reticulum kinase; TRB3, tribbles 3; UPR, unfolded protein response; Xbp1, X-box binding protein 1. The figure was created by authors using BioRender.com on 21 March 2023.

On the other hand, oxidative stress is known to cause ERS by directly causing oxidative damage to proteins, thereby compromising the ER protein folding process and causing the accumulation of unfolded or misfolded proteins [84,85]. In addition, reactive oxygen species also cause ERS by promoting ER calcium release [86,87]. Tocotrienol is a known antioxidant, but it may exert pro-oxidant activities at high concentrations [88]. The role of the pro-oxidant properties of tocotrienols in triggering ERS is not elucidated. The only related study reported that the concentration of γ-tocotrienol in inducing ERS did not cause the pro-oxidant effects [47]. Interestingly, 6-O-carboxypropyl-alpha-tocotrienol, a semi-synthesized tocotrienol, also serves as an ERS inducer [89]. This semi-synthesized tocotrienol induced ERS by inhibiting proteasome activity and/or downregulating proteasome complex protein subunit beta-type (PSMB) 1-6 levels [89], which eventually led to the accumulation of polyubiquitinated proteins in the ER lumen. Mechanistically, this semi-synthesized tocotrienol inhibited transcription factors such as nuclear factor erythroid 2 related factor-1 (NRF1) and STAT3 transcription factors, which subsequently downregulated PSMB proteins [89]. A similar finding was reported by Ramdas et al., whereby a proteomic analysis revealed a significant downregulation of PSMB1, PSMB6, and seven other subtypes of proteasome complex proteins upon γ-tocotrienol treatment [90]. Future studies will emphasize the mechanisms of tocotrienol-upregulated ER calcium release, ceramide synthesis, proteasomal inhibition, and protein folding status. The current findings on the molecular mechanisms of tocotrienol in inducing ERS and ERS-related cell fate are summarized in Figure 4.

### 4.5. ERS-Inducing Properties of Vitamin E Analogues

On the other hand, tocopherol [45,47,55,91,92], another isoform of vitamin E, and α-tocopheryl succinate (vitamin E succinate, a semi-synthetic tocopherol) [93,94] also demonstrated ERS-inducing properties on several cancer cells. In terms of potency, tocopherol was a less potent ERS inducer than tocotrienol, whereby a 4 to 8 times higher concentration of tocopherol is needed to induce a similar ERS outcome as tocotrienol [45]. TRF from palm oil that consists of a mixture of 32% α-tocopherol, 25% α-tocotrienol, 29% γ-tocotrienol, and 14% δ-tocotrienol was also not as potent as an equimolar purified γ- or δ-tocotrienol in inducing ERS [50]. This could be due to the suboptimal concentration of those active ERS-inducing isoforms and the potential interference between isoforms, including tocopherol, in inducing ERS. The interaction between isoforms, including tocopherol, has not been determined so far. An antagonistic relationship between α-tocopherol and tocotrienol was reported [95,96]. However, a combination of γ- and δ-tocotrienol [97], γ-tocopherol and annatto tocotrienol [98], or γ-tocopherol and γ-tocotrienol [92] was also reported to significantly improve the anticancer properties. Therefore, it is important to identify the optimal spectrum of tocotrienol isomers that exert the best anticancer effects. Although the use of pure isomers is effective, the purification process could be costly.

### 4.6. Limitations

There are several limitations to this systematic review. The reproducibility of data is of concern, whereby some of the included studies did not disclose the tocotrienol stock solution preparation [48,52] and demonstrate the solvent or vehicle effect [43,49,50,51,52,53]. In addition, the tocotrienol stock solution preparation varies across the studies. Our group and other research teams employed overnight serum incubation, where serum contains albumin and transfer proteins that could enhance the cellular uptake of tocotrienol, which is highly lipophilic [29,31,99,100,101,102,103,104,105,106]. Similarly, the use of bovine serum albumin in dissolving tocotrienol also improved its cellular uptake [43,49,107]. The relevant study for investigating and comparing the differential effect of stock preparation, including serum incubation, on tocotrienol is not available and should be investigated. Furthermore, some of the included articles did not disclose the purity of the tocotrienol used [44,45,47,48]. Most of the studies used a high purity of tocotrienol (≥95%), but interference from minor components such as carotenoids, flavonoids, and plant pigments cannot be ruled out. Several included studies [49,51,52] only examined the total IRE1α or PERK levels without their phosphorylated forms, which could cause interpretation bias. Moreover, the biological variations among different cancer cell models cannot be neglected. Additionally, non-English, unpublished, and gray literature were not considered. Conference abstracts and theses were not included as they might overlap with published articles. Furthermore, UPR markers and ERS-related apoptosis markers were not included as keywords in the literature search. To overcome these shortcomings, we checked the reference lists of the included studies to identify any additional and relevant studies.

## 5. Conclusions

The currently available evidence has consistently demonstrated ERS-inducing properties of tocotrienols via the upregulation or activation of ERS sensors or UPR markers. Tocotrienol-induced ERS was crucial in the subsequent ERS-mediated apoptosis. Nevertheless, the upstream molecular mechanisms of tocotrienols in triggering ERS are largely unknown. Several events, such as ER calcium release, ceramide and/or dihydroceramide upregulation, proteasomal inhibition, and miR-190b upregulation, were suggested to serve as the early events in modulating or triggering the ERS. However, the protein folding status and the precise mechanism of action of tocotrienol have not been determined. Further studies are required to elucidate the molecular mechanism and the upstream molecular targets in tocotrienol-mediated ERS, wherein these mechanistic findings will be essential in developing tocotrienol or chemical derivatives in personalized medicine.

## Data Availability

Not applicable.

## Supplementary Materials

The following supporting information can be downloaded at: https://www.mdpi.com/article/10.3390/nu15081854/s1. Supplementary Table S1: Search strategy of this systematic review [108]; Supplementary Table S2: PRISMA checklist of this systematic review; Supplementary Table S3: OHAT risk-of-bias tool for in vitro studies.
