# Supplementary material for: Molecular Mechanism of Tocotrienol-Mediated Anticancer Properties: A Systematic Review of the Involvement of Endoplasmic Reticulum Stress and Unfolded Protein Response
Source: Nutrients. 2023 Apr 12;15(8):1854. doi: 10.3390/nu15081854 (PMC10145773; doi:10.3390/nu15081854)
Supplement: Supplementary file 1 [file nutrients-15-01854-s001.zip › nutrients-2330385-supplementary/Supplemetary Table S2.pdf]

Supplementary Table S2. Search strategy of this systematic review

Database	Search strategy
PubMed	((“vitamin E”) OR (tocotrienol) OR (tocopherol)) AND (("endoplasmic reticulum stress") OR (ERS) OR ("unfolded protein response") OR (UPR))
WoS	((“vitamin E”) OR (tocotrienol) OR (tocopherol)) AND (("endoplasmic reticulum stress") OR (ERS) OR ("unfolded protein response") OR (UPR)); all field
Scopus	TITLE-ABS-KEY((“vitamin E”) OR (tocotrienol) OR (tocopherol)) AND (("endoplasmic reticulum stress") OR (ERS) OR ("unfolded protein response") OR (UPR)); search title, abstract, keywords, article only, limited to English and research articles
EMBASE	((“vitamin E”) OR (tocotrienol) OR (tocopherol)) AND (("endoplasmic reticulum stress") OR (ERS) OR ("unfolded protein response") OR (UPR)); database from 1988 to week 7, 2023
