# Supplementary material for: Molecular Mechanism of Tocotrienol-Mediated Anticancer Properties: A Systematic Review of the Involvement of Endoplasmic Reticulum Stress and Unfolded Protein Response
Source: Nutrients. 2023 Apr 12;15(8):1854. doi: 10.3390/nu15081854 (PMC10145773; doi:10.3390/nu15081854)
Supplement: Supplementary file 1 [file nutrients-15-01854-s001.zip › nutrients-2330385-supplementary/Supplemetary Table S3.pdf]

Supplementary Table S3. OHAT risk-of-bias tool for in vitro studies

No.	OHAT non-human studies domains	Included studies										
		Wali et al. [1]	Park et al. [2]	Gopalan et al. [3]	Patacsil et al. [4]	Xiong et al. [5]	Tuerdi et al. [6]	Tiwari et al. [7]	Comitato et al. [8]	Marelli et al. [9]	Fontana et al. [10]	Ambra et al. [11]
1	Was administered dose or exposure level adequately randomized??	++	++	++	++	++	++	++	++	++	++	++
2	Was allocation to study groups adequately concealed?	++	++	++	++	++	++	++	++	++	++	++
3	Were experimental conditions identical across study groups?	++	++	++	++	++	++	++	++	++	++	++
4	Were research personnel blinded to the study group during the study?	-*	-*	-*	-*	-*	-*	-*	-*	-*	-*	-*
5	Were outcome data complete without attrition or exclusion from analysis?	++	++	++	++	++	++	++	++	++	++	++
6	Can we be confident in the exposure characterization?	+#	++	++	++	++	+**	+#	+#	+#	-**, #	+#
7	Can we be confident in the outcome assessment?	++	++	++	++	++	++	++	++	++	++	++
8	Were all measured outcomes reported?	++	++	++	++	++	++	++	++	++	++	++
9	Were there no other potential threats to internal validity?	++	++	++	++	++	++	++	++	++	++	-##
	**Overall appraisal: (Tier 1, Tier 2 or Tier 3)**	**1**	**1**	**1**	**1**	**1**	**1**	**1**	**1**	**1**	**1**	**1**

Abbreviations:
++, Definitely low risk of bias; +, Low risk of bias; -, Probably high risk; - - Definitely high risk

Notes:
* The authors did not mention about experimental blinding; ** The authors did not disclose tocotrienol stock preparation or storage
# The authors did not state the maximal concentration of tested solvent (vehicle control); ## The authors did not show the comparative control's result.
Adapted from OHAT Risk of Bias Rating Tool for Human and Animal Studies and emphasizes on 9 domains that relevant to *in vitro* studies [12,13]. Tier grouping was based on a previous study [13].

References:

1. Wali, V.B.; Bachawal, S.V.; Sylvester, P.W. Endoplasmic reticulum stress mediates gamma-tocotrienol-induced apoptosis in mammary tumor cells. *Apoptosis* **2009**, *14*, 1366-1377, doi:10.1007/s10495-009-0406-y.
2. Park, S.K.; Sanders, B.G.; Kline, K. Tocotrienols induce apoptosis in breast cancer cell lines via an endoplasmic reticulum stress-dependent increase in extrinsic death receptor signaling. *Breast Cancer Res Treat* **2010**, *124*, 361-375, doi:10.1007/s10549-010-0786-2.
3. Gopalan, A.; Yu, W.P.; Jiang, Q.; Jang, Y.M.; Sanders, B.G.; Kline, K. Involvement of de novo ceramide synthesis in gamma-tocopherol and gamma-tocotrienol-induced apoptosis in human breast cancer cells. *MOLECULAR NUTRITION & FOOD RESEARCH* **2012**, *56*, 1803-1811, doi:10.1002/mnfr.201200350.
4. Patacsil, D.; Tran, A.T.; Cho, Y.S.; Suy, S.; Saenz, F.; Malyukova, I.; Ressom, H.; Collins, S.P.; Clarke, R.; Kumar, D. Gamma-tocotrienol induced apoptosis is associated with unfolded protein response in human breast cancer cells. *J Nutr Biochem* **2012**, *23*, 93-100, doi:10.1016/j.jnutbio.2010.11.012.
5. Xiong, A.; Yu, W.; Tiwary, R.; Sanders, B.G.; Kline, K. Distinct roles of different forms of vitamin E in DHA-induced apoptosis in triple-negative breast cancer cells. *Mol Nutr Food Res* **2012**, *56*, 923-934, doi:10.1002/mnfr.201200027.
6. Tuerdi, G.; Ichinomiya, S.; Sato, H.; Siddig, S.; Suwa, E.; Iwata, H.; Yano, T.; Ueno, K. Synergistic effect of combined treatment with gamma-tocotrienol and statin on human malignant mesothelioma cells. *Cancer Lett* **2013**, *339*, 116-127, doi:10.1016/j.canlet.2013.07.015.
7. Tiwari, R.V.; Parajuli, P.; Sylvester, P.W. γ-Tocotrienol-induced endoplasmic reticulum stress and autophagy act concurrently to promote breast cancer cell death. *Biochem Cell Biol* **2015**, *93*, 306-320, doi:10.1139/bcb-2014-0123.
8. Comitato, R.; Guantario, B.; Leoni, G.; Nesaretnam, K.; Ronci, M.B.; Canali, R.; Virgili, F. Tocotrienols induce endoplasmic reticulum stress and apoptosis in cervical cancer cells. *Genes Nutr* **2016**, *11*, 32, doi:10.1186/s12263-016-0543-1.
9. Marelli, M.M.; Marzagalli, M.; Moretti, R.M.; Beretta, G.; Casati, L.; Comitato, R.; Gravina, G.L.; Festuccia, C.; Limonta, P. Vitamin E delta-tocotrienol triggers endoplasmic reticulum stress-mediated apoptosis in human melanoma cells. *SCIENTIFIC REPORTS* **2016**, *6*, doi:10.1038/srep30502.
10. Fontana, F.; Moretti, R.M.; Raimondi, M.; Marzagalli, M.; Beretta, G.; Procacci, P.; Sartori, P.; Montagnani Marelli, M.; Limonta, P. δ-Tocotrienol induces apoptosis, involving endoplasmic reticulum stress and autophagy, and paraptosis in prostate cancer cells. *Cell Prolif* **2019**, *52*, e12576, doi:10.1111/cpr.12576.
11. Ambra, R.; Manca, S.; Leoni, G.; Guantario, B.; Canali, R.; Comitato, R. Involvement of miR-190b in Xbp1 mRNA Splicing upon Tocotrienol Treatment. *Molecules* **2020**, *26*, doi:10.3390/molecules26010163.
12. Office of Health Assessment and Translation. Risk of bias tool. Available online: https://ntp.niehs.nih.gov/whatwestudy/assessments/noncancer/riskbias/index.html (accessed on March 15 2023).
13. Romeo, S.; Zeni, O.; Sannino, A.; Lagorio, S.; Biffoni, M.; Scarfi, M.R. Genotoxicity of radiofrequency electromagnetic fields: Protocol for a systematic review of in vitro studies. *Environ Int* **2021**, *148*, 106386, doi:10.1016/j.envint.2021.106386.
